# Let-7a-transfected mesenchymal stem cells ameliorate monocrotaline-induced pulmonary hypertension by suppressing pulmonary artery smooth muscle cell growth through STAT3-BMPR2 signaling

**DOI:** 10.1186/s13287-017-0480-y

**Published:** 2017-02-10

**Authors:** Gesheng Cheng, Xingye Wang, Yongxin Li, Lu He

**Affiliations:** 1 Department of Cardiology, the First Affiliated Hospital of Xi’an Jiaotong University, Xi’an, 710061 People’s Republic of China; 2 Department of Cardiovascular Surgery, the First Affiliated Hospital of Xi’an Jiaotong University, Xi’an, 710061 People’s Republic of China

**Keywords:** Pulmonary arterial hypertension, mesenchymal stem cells, let-7a, pulmonary artery smooth muscle cell, STAT3 pathway, BMPR2

## Abstract

**Background:**

Cell-based gene therapy has become a subject of interest for the treatment of pulmonary arterial hypertension (PAH), a devastating disease characterized by pulmonary artery smooth muscle cell (PASMC) hyperplasia. Mesenchymal stem cells (MSCs) have been recently acknowledged as a potential cell vector for gene therapy. Here, we investigated the effect of MSC-based let-7a for PAH.

**Methods:**

After isolation and identification of MSCs from rat bone marrow, cells were infected with recombinant adenovirus vector Ad-let-7a. Lewis rats were subcutaneously injected with monocrotaline (MCT) to induce PAH, followed by the administration of MSCs, MSCs-NC (miR-control), or MSC-let-7a, respectively. Then, right ventricular systolic pressure (RVSP), right ventricular hypertrophy, and pulmonary vascular remodeling were evaluated. Rat pulmonary artery smooth muscle cells (rPASMCs) under hypoxia were co-cultured with MSCs or MSC-let-7a. Cell proliferation and apoptosis were separately determined by ^3^H thymidine incorporation and flow cytometry analysis. The underlying mechanism was also investigated.

**Results:**

MSC transplantation enhanced let-7a levels in MCT-induced PAH rats. After injection with MSC-let-7a, RVSP, right ventricular hypertrophy, and pulmonary vascular remodeling were notably ameliorated, indicating a protective effect of MSC-let-7a against PAH. When co-cultured with MSC-let-7a, hypoxia-triggered PASMC proliferation was obviously attenuated, concomitant with the decrease in cell proliferation-associated proteins. Simultaneously, the resistance of PASMCs to apoptosis was remarkably abrogated by MSC-let-7a administration. A mechanism assay revealed that MSC-let-7a restrained the activation of signal transducers and activators of transcription 3 (STAT3) and increased its downstream bone morphogenetic protein receptor 2 (BMPR2) expression. Importantly, preconditioning with BMPR2 siRNA dramatically abated the suppressive effects of MSC-let-7a on PASMC proliferation and apoptosis resistance.

**Conclusions:**

Collectively, this study suggests that MSCs modified with let-7a may ameliorate the progression of PAH by inhibiting PASMC growth through the STAT3-BMPR2 signaling, supporting a promising therapeutic strategy for PAH patients.

## Background

Pulmonary arterial hypertension (PAH) is a well-recognized, devastating, and life-threatening disease. As a common progressive disease, it is characterized by a sustained increase in pulmonary arterial pressure, right heart dysfunction, and vascular remodeling, leading to the high morbidity and mortality of patients with lung and heart diseases [1–3]. Despite therapeutic advances in PAH treatment, the 3-year survival rate is still less than 60% [4]. Therefore, an understanding of the molecular mechanism underlying PAH development is urgently required for improving the therapeutic strategies.

Recently, cell-based gene therapies have garnered increasing interest due to their beneficial roles in ameliorating the progression of PAH [5–7]. Mesenchymal stem cells (MSCs) are the preferred cell model for cell-based therapy because of their high expansion in culture, reproducible potential, and capacity to differentiate into various lineages. They can be mobilized and migrate into injury sites to orchestrate the repair response [8]. Application of naïve MSCs attenuates monocrotaline (MCT)-induced PAH, indicating a beneficial effect of stem cell therapy for PAH disorders [9, 10]. More interestingly, administration of MSCs modified with prostacyclin synthase or endothelial nitric oxide synthase not only abrogated right ventricular (RV) hypertrophy and cardiovascular remodeling, but also improved survival time in PAH rats [5, 6]. Additionally, MSCs expressing heme oxygenase-1 enhanced the protective effect of MSCs on hypoxia-triggered PAH by attenuating lung inflammation and smooth muscle cell proliferation, suggesting a promising strategy against PAH based on MSC-based gene therapy [11].

MicroRNAs (miRNAs) orchestrate various pathophysiological processes including lipid metabolism, vascular remodeling, and tissue repair [12–15]. Abnormal expression of miRNAs has been corroborated in several lung-related diseases, such as PAH [16]. The presence of some miRNAs that are correlated with PAH severity can retard the pathological progression of PAH, indicating a potential innovative therapy for the treatment of PAH [17]. Animals with PAH show a dramatic downregulation of let-7a in the lungs [16]. An analogous decrease in let-7a was also validated when cells were exposed to chronic hypoxia, suggesting a potential role of let-7a in the development of PAH [18]. Moreover, let-7 expression also negatively correlated with the severity of PAH in patients with systemic scleroderma [19]. Unfortunately, its function in PAH remains poorly defined. In this study, we aimed to explore the function of MSCs transfected with let-7a in PAH development. Furthermore, the effects of MSC-let-7a on pulmonary artery smooth muscle cell (PASMC) growth, as well as the underlying mechanism, were also explored.

## Methods

### Antibodies and reagents

The signal transducer and activator of transcription 3 (STAT3) inhibitor, S31-201, was acquired from Sigma (St. Louis, MO, USA). The primary antibodies against STAT3, phosphorylated STAT3-Tyr-705 (p-STAT3), proliferating cell nuclear antigen (PCNA), and bone morphogenetic protein receptor 2 (BMPR2) were purchased from Abcam (Cambridge, UK). Rabbit antibodies against rat p21 and α-smooth muscle actin (SMA) were obtained from Sigma. Anti-rat antibodies of Ki67 and cyclin D1 were purchased from Santa Cruz Biotechnology (Santa Cruz, CA, USA).

### Animals

For experiments using animals, 6-week-old male Lewis rats were purchased from Beijing Vital River Laboratory Animal Technology Co., Ltd. (Beijing, China). All animal care and experimental procedures were undertaken with approval from the Institutional Animal Care and Use Committee of the First Affiliated Hospital of Xi’an Jiaotong University (SCXK-2012-003). Rats were acclimatized for 1 week prior to the subsequent treatments.

### Isolation and expansion of rat MSCs

Preparation of rat MSCs (rMSCs) was conducted following previously published methods [6]. Briefly, bone marrow cells from Lewis rats were mechanically dissociated by flushing the femur and tibia cavities under sterile conditions. The isolated cells were then suspended in α-MEM medium containing 10% fetal bovine serum (FBS), 100 U/mL penicillin and 100 μg/mL streptomycin on 75-cm^2^ tissue culture flasks. Three days later, non-adherent cells were removed, and the adherent cells that had grown to 90% confluence were considered as passage (P)0 MSCs. For expansion, cells were diluted in the ratio of 1:2 per passage and continuously cultured in rMSC medium. Passages 3–4 were used for the subsequent experiments.

### Characterization of MSCs in vitro

The surface marker proteins of MSCs were identified by incubation with fluorescein isothiocyanate (FITC)-conjugated monoclonal antibodies against CD34, CD45, CD44, and CD90 (all from Abcam, Cambridge, MA, USA). Then, the labeled cells were analyzed by a FACScan flow cytometer (Becton Dickinson, NJ, USA). To evaluate osteogenic ability of MSCs, MSCs of P3 were incubated with osteogenic medium for 4 weeks and subsequently stained with 2% Alizarin Red S (Sigma). Cells were stained with 0.3% Oil Red O (Sigma) to assess adipogenic ability. All specimens were analyzed microscopically (IX70, Olympus, Tokyo, Japan).

### Adenovirus production for let-7a in rMSCs

Adenoviral vectors of AdmiRa-rno-let-7a expressing green fluorescent protein (GFP) and miR-control (normal control; NC) were obtained from Applied Biological Materials Inc. (Richmond, BC, Canada). The above adenovirus was packaged according to the protocol provided by the manufacturers. After amplification and purification, virus titers were measured by the p24 ELISA kit (Cell Biolabs, Inc., San Diego, CA, USA). Then, the obtained rMSCs at P3–P4 were infected with the above adenovirus to generate the let-7a-overexpressed MSCs. The infection efficiency was evaluated by GFP and quantitative RT-PCR. Cell that overexpressed let-7a were then harvested with phosphate-buffered saline (PBS) buffer supplement with 0.25% trypsin and 1 mmol/L EDTA before use.

### PAH model

To construct the PAH model, Lewis rats received a subcutaneous injection of monocrotaline (MCT; Sigma, St. Louis, MO, USA; 60 mg/kg) as previously reported [6]. Rats injected with 0.9% saline were defined as the negative control.

### Animal groups

About 3 weeks later, rats treated with MCT were randomized to four groups (*n* = 10/group, *n* = 40 in total): rats treated with PBS (MCT + PBS group); rats received MSCs (MCT + MSC group); rats injected with MSC-let-7a (MCT + MSC-let-7a group); rats received MSC-NC (MCT + MSC-NC group). For cell implantation, approximately 3 × 10^6^ cells in PBS were slowly injected into rats via the tail vein. Rats without MCT administration were defined as the control group.

### Collection of blood specimens

EDTA-anticoagulated whole blood samples were collected at 7, 14, and 21 day after the injection of MSC-let-7a, MSCs, or PBS. The bloods were centrifuged at 1000 g for 10 min. The obtained specimens were then stored at –70 °C until analysis.

### Hemodynamic evaluation and right ventricular hypertrophy

Three weeks after MSC administration, rats were anesthetized with sodium pentobarbital (30 mg/kg) to perform the hemodynamic study. The 3-F Miller catheters were inserted into the right ventricle through the right jugular vein to assess the right ventricular systolic pressure (RVSP). After euthanizing with an overdose of pentobarbital, the lungs and hearts were collected. The ratios of right ventricle weight to body weight (RVW/BW) and right ventricle/left ventricle + septum weight (RV/LV + Sep) were detected and defined as the indexes of right ventricular hypertrophy [5, 6]. For vascular remodeling evaluation, lung tissues were embedded in paraffin and then were cut into serial 4-μm thick sections. After staining with hematoxylin and eosin, the wall thickness (*n* = 5 measurements/rat in 10 rats) was calculated as follows: wall thickness (%) = (external diameter – internal diameter)/ external diameter × 100 [7].

### Cell culture

Rat pulmonary artery smooth muscle cells (rPASMCs) were purchased from Pricells Company (Wuhan, China) and cultured in DMEM medium containing 5% FBS, 100 U/ml penicillin, and 100 μg/ml streptomycin. Cells from P5–P10 were used for the subsequent experiments. All cells were maintained in 5% CO_2_ at 37 °C.

### Knockdown of BMPR2 by siRNA

To knockdown BMPR2 expression in MSCs, three specific pairs of siRNA sequences were designed according to rat BMPR2 mRNA (GenBank accession number: NM_080407.1). Their sequences were as follows: siRNA-BMPR2-1500 (sense, 5′-GGUUGGCACAAUCCGCUAU-3′; antisense, 5′-AUAGCGGAUUGUGCCAACC-3′), siRNA-BMPR2-1947 (sense, 5′-GCAGAACGAACGCAACCUA-3′; antisense, 5′-UAGGUUGCGUUCGUUCUGC-3′), and siRNA-BMPR2-2173 (sense, 5′-CCAGAAACAAGCGUCACAA-3′; antisense, 5′- UUGUGACGCUUGUUUCUGG-3′). The scramble siRNA (siRNA-NC) were used as negative control, and the sequences were as follow: forward 5′-GGUCACGCUAACGCGUUAU-3′, reverse 5′-AUAACGCGUUAGCGUGACC-3′. For transfection, cells were grown to approximately 50–60% confluence. Then, cells were transfected with siRNAs at 4 nM using Lipofectamine 2000 (Invitrogen, Carlsbad, CA, USA) according to the manufacturer's directions. Approximately 24 h later, the efficiency of BMPR2 siRNA was analyzed by Western blotting.

### Co-culture of MSCs and rPASMCs

MSCs and PASMCs were co-cultured using the transwell system as previously described [20]. Briefly, rPASMCs were exposed to hypoxia with 3% oxygen concentration or normoxia for 24 h. Cells were then seeded in the transwell insert membrane with 0.4-mm diameter pores. Then, the modified MSCs (MSC-NC or MSC-let-7a) were cultured in the lower chamber. Twenty-four hours later, the PASMCs were collected for cell proliferation and apoptosis assay.

### RNA extraction and quantitative RT-PCR

Total RNA from blood samples was obtained by the RNAprep Pure Hi-Blood Kit (TIANGEN, Beijing, China) according to the manufacturer’s directions. Total RNA from lung tissues and cells was extracted using TRIzol reagent (Invitrogen Life Technologies, Carlsbad, CA, USA). For mRNA detection, reverse transcription was performed to synthesize the cDNA with RevertAidTM First Strand cDNA Synthesis Kits (Fermentas, St. Leon-Rot, Germany). Then, quantitative RT-PCR was carried out to evaluate the mRNA levels of let-7a using SYBR premix Ex Taq (TaKaRa) according to the manufacturer’s protocol. The specific primers for let-7a, Ki67, cyclinD1, PCNA, and fibronectin (Fn) were used as described previously [21–23]. The expression of let-7a was normalized to U6, and β-actin was used as a quality control to normalize other gene expression. All data were calculated with the 2^–ΔΔCt^ equation.

### Western blotting

The collected tissues and cells were lysed with RIPA lysis buffer (Thermo scientific, Rockford, IL, USA) followed by the detection of protein concentration using the BCA assay kit (Pierce, Rockford, IL, USA). Then, equal amounts of samples were subjected to 12% SDS–PAGE, and the separated proteins were electro-blotted onto PVDF membranes (Sigma), which were blocked with 5% nonfat dry milk in TBST for 2 h. The membranes were then incubated at room temperature with the primary antibodies against Ki67, cyclin D1, p21, PCNA, and α-SMA. The HRP-conjugated secondary antibodies were then added for further incubation for 1 h. The binding proteins were visualized using chemiluminescence (Santa Cruz, CA, USA) and quantified by a Gel DocTM XR imaging system (Bio-Rad Laboratories, Hercules, CA, USA).

### Cell proliferation assay

PASMC proliferation was assessed using ^3^H thymidine (^3^H-TdR; Atomic Nucleus Research Institute, Shanghai, China) incorporation in accordance with the manufacturer’s instructions. Briefly, 37 kBq/ml of ^3^H-TdR was added to the co-culture system. Twenty-four hours later, cell viability was analyzed by detecting radioactivity with a liquid scintillation counter (Beckmen, USA). At least three independent experiments were performed to evaluate cell viability.

### Flow cytometry analysis

Following co-incubation with MSCs for 24 h, the PASMCs were collected. Then, cells were trypsinized and suspended with binding buffer. About 10 μl Annexin V-FITC and 5 μl PI were added for further incubation at room temperature. Fifteen minutes later, all specimens were subjected to a FACScan flow cytometer (Becton Dickinson, NJ) to analyze the relative apoptosis via at least three independent repeats.

### Caspase-3 activity

Caspase-3 activity was performed using the Caspase-Glo 3/7 Assay kit (Promega, Madison, WI, USA). Following the co-culture with MSC-NC or MSC-let-7a, caspase-3 activity was detected according to the manufacturer’s instructions. The results were obtained from three independent experiments.

### Statistical analysis

All experiments were conducted independently at least three times and all results are shown as mean ± SD. The unpaired Student’s *t* test was used to analyze the statistical significance of differences between two groups. Comparisons among three or more groups were made with ANOVA followed by the post-hoc test, and *p* < 0.05 was considered statistically significant.

## Results

### Characterization of MSCs isolated from bone marrow

**Fig. 1:**
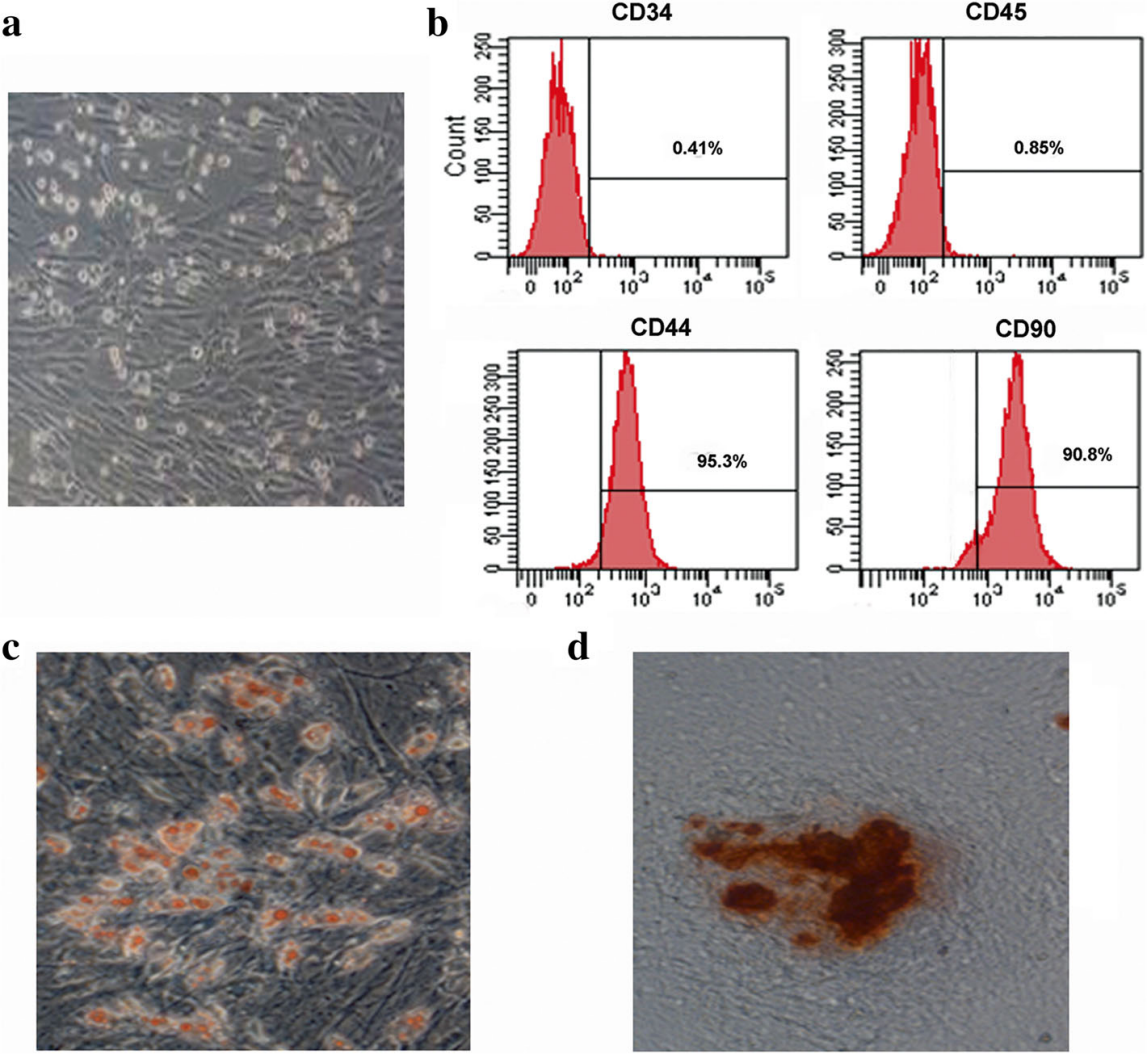
Characterization of MSCs. **a** Morphological characterization of MSCs at passage 3. **b** Flow cytometry analysis suggested the major phenotype characteristics of MSCs (CD34^−^, Cd45^−^, CD44^+^, and CD90^+^). **c** To evaluate osteogenic ability of MSCs, cells were stained with 2% Alizarin Red S. The Alizarin Red S-positive mineral deposits were observed in MSCs. **d** Cells were stained with 0.3% Oil Red O to assess their adipogenic ability. Analysis with a microscope confirmed the accumulation of lipids in MSCs. Magnification × 200

As shown in Fig. 1a, most MSCs at P3 were spindle-like in shape and adhered to the plastic tissue culture dishes after culture for 7 days. Moreover, flow cytometric assay confirmed that MSCs were stained negatively for the hematopoietic markers CD34 and CD45, but highly expressed the mesenchymal associated markers CD44 and CD90 (Fig. 1b). To further evaluate the multipotency of MSCs differentiating into osteogenic and adipogenic cells, MSCs were incubated in osteogenic and adipogenic medium. As expected, MSCs could form alizarin-red-positive mineral deposits (Fig. 1c). Furthermore, MSCs exhibited an adipogenic potential by detecting lipid droplet deposition through Oil Red O staining (Fig. 1d). Therefore, all data suggested that we successfully isolated MSCs with stemness.

### Expression levels of let-7a in MSCs

**Fig. 2:**
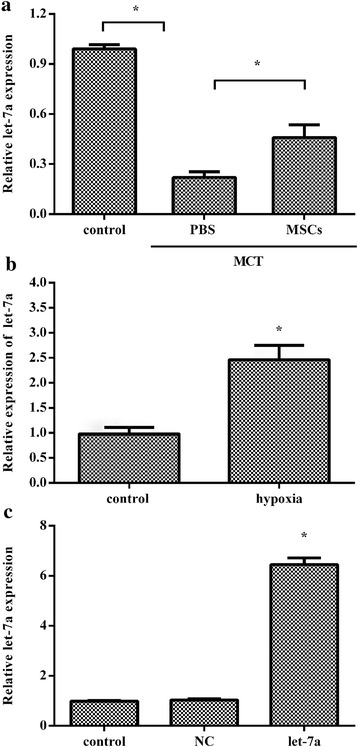
Expression of let-7a in MSCs and PAH rats. Rats were injected with MCT (60 mg/kg) for 2 weeks to induce the PAH model. Then, PBS and MSCs were injected into rats with PAH. The expression of let-7a was analyzed by quantitative RT-PCR (**a**). MSCs were exposed to hypoxia (3% oxygen) or normoxia (control group) for 24 h. The expression of let-7a was detected in control and hypoxia-treated groups (**b**). To explore the effects of MSC-based therapy against PAH, MSCs were exposed to Ad-let-7a or Ad-NC viruses and cultured for 48 h. Then, quantitative RT-PCR was performed to detect let-7a expression in MSCs (**c**). *p < 0.05

Consistent with a previous report [16], an obvious attenuation of let-7a expression was substantiated in PAH rats (Fig. 2a). Engraftment of MSCs dramatically ameliorated the downregulation of let-7a expression in PAH animals, indicating a potential critical role of let-7a in the development of PAH. Furthermore, a higher expression of let-7a was also conferred in MSCs exposed to hypoxia, in contrast to the normoxia group (Fig. 2b). To investigate the effect of MSCs modified with let-7a on PAH development, MSCs were infected with the recombinant Ad-let-7a or Ad-NC. Quantitative RT-PCR assay showed that the expression of let-7a in let-7a^+^ MSCs was obviously enhanced relative to the control group (Fig. 2c).

### Transplantation of MSC-let-7a ameliorates MCT-triggered right ventricular impairment

**Fig. 3:**
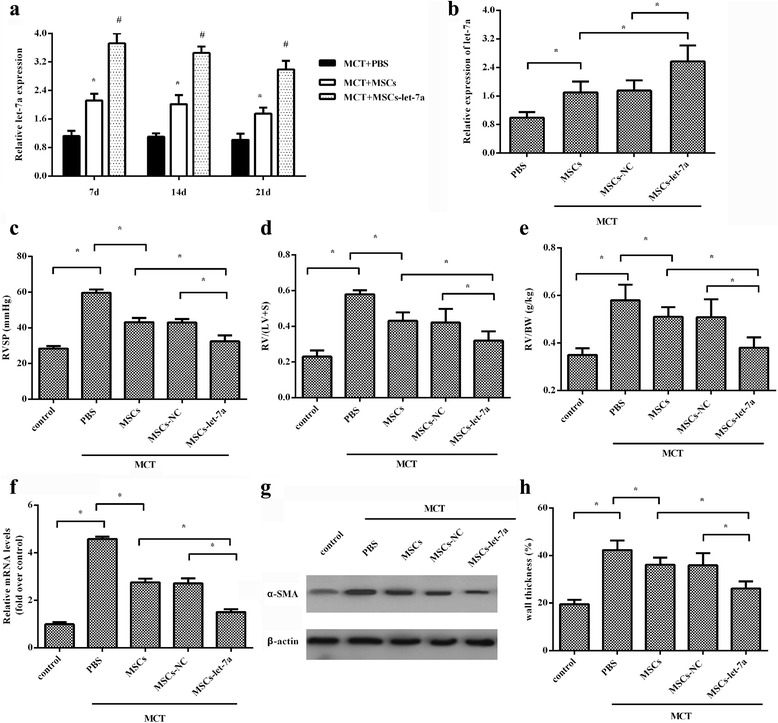
Effect of MSC-let-7a on the development of PAH. Rats were given PBS, MSCs, MSC-NC, or MSC-let-7a 2 weeks after intraperitoneal injection with MCT. The blood samples were collected at 7, 14, and 21 days after injection. The expression of let-7a was analyzed by quantitative RT-PCR (**a**) and, about 3 weeks later, the expression of let-7a was analyzed in lung tissues (**b**). The effect of MSC-let-7a on RVSP was analyzed by inserting 3-F Miller catheters into the right ventricle through the right jugular vein (**c**). Then, rats were euthanized and the hearts and lung tissues were harvested. The ratios of RV/LV + S (**d**) and RV/BW (**e**) were determined to evaluate right ventricular hypertrophy. Following lysis with RIPA buffer, the mRNA levels of Fn in lung tissues were detected by quantitative RT-PCR (**f**). The expression of α-SMA was analyzed by Western blotting (**g**). Lung tissues were embedded in paraffin, and then were cut into serial 4-μm thick sections. Wall thickness was then evaluated by HE staining (**h**). *p < 0.05. *BW* body weight, *LV* left ventricle, *MCT* monocrotaline, *MSC* mesenchymal stem cell, *NC* miR-control, *PBS* phosphate-buffered saline, *RV* right ventricle, *RVSP* right ventricular systolic pressure, *S* septum, *SMA* smooth muscle actin

To explore the efficacy of MSC-based let-7a elevation in reversing MCT-triggered PAH, the constructed MSC-let-7a were injected into rats with MCT treatment. In contrast to the PBS group, the expression of let-7a was obviously increased at 7 days after injection with MSCs, which was further elevated in the MSC-let-7a group. Moreover, 3 week later, the expression of let-7a was still maintained at a relatively higher level in MSC-let-7a groups relative to PBS and MSC groups (Fig. 3a). Furthermore, the expression of let-7a in lung tissues was also increased in MSC-let-7a groups (Fig. 3b). Additionally, rats that received MCT treatment provoked a strong RVSP, which was obviously attenuated by MSC treatment (Fig. 3c). Interestingly, MSC-let-7a administration further reduced RVSP in contrast to MSC-treated groups. Importantly, MSC-let-7a transplantation strikingly antagonized the MCT-induced increase in the ratio of RV/(LV + Sep), an index of right ventricular hypertrophy (RVH), relative to the MSC-treated groups, but without a statistically significant difference between normal MSC and NC-MSC groups (Fig. 3d). Consistently, the increase in the ratio of RV/BW induced by MCT, another index of RVH, was also obviously decreased following MSC-let-7a treatment (Fig. 3e). These results indicate a potential protective effect of MSC-let-7a on PAH development.

### Treatment with let-7a/MSCs attenuates pulmonary vascular remodeling induced by MCT

Pulmonary vascular remodeling is the major characterization of PAH with hyperplasia of PASMCs and deposition of extracellular matrix (ECM) [1, 7]. To further clarify the effect of MSC-let-7a on MCT-induced pulmonary vascular remodeling, the expression of ECM protein fibronectin (Fn) was determined. As shown in Fig. 3f, MCT induced an approximately 4.57-fold increase in Fn mRNA levels in the lung, which was notably attenuated following MSC treatment. Moreover, a more obvious decrease in Fn expression was seen in the MSC-let-7a groups than that in the MSC groups. Specifically, the elevated expression of α-SMA, a marker for SMCs that is used to evaluate muscularization of pulmonary arteries, was obviously restrained following injection with MSC-let-7a (Fig. 3g). Importantly, the notable increase in wall thickness of pulmonary arterioles was markedly restrained when rats received MSC-let-7a administration (Fig. 3h). In brief, these data suggested that MSC-let-7a might participate in MCT-induced pulmonary vascular remodeling.

### Overexpression of let-7a in MSCs regulates hypoxia-induced PASMC proliferation

**Fig. 4:**
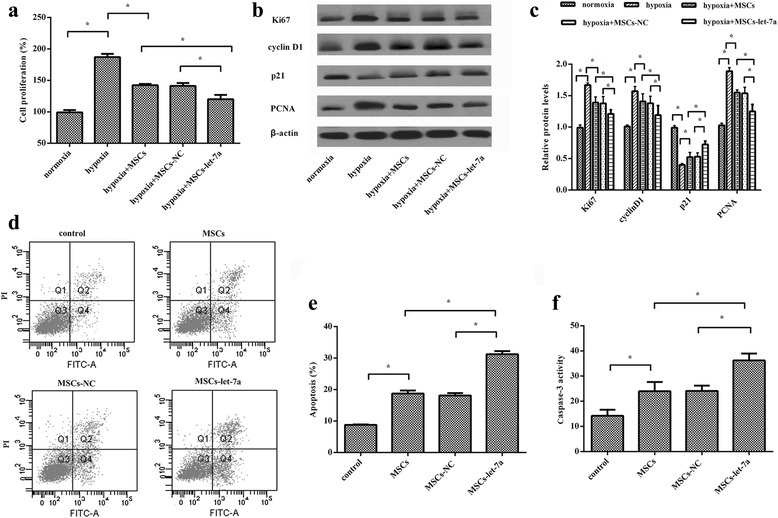
MSC-let-7a administration dramatically inhibited PASMC proliferation and reduced its resistance to apoptosis. The rPASMCs were exposed to hypoxia or normoxia for 24 h, followed by co-culture with modified MSCs (MSC-NC or MSC-let-7a) using the transwell system. Approximately 24 h after incubation, PASMCs were collected. Cell viability was analyzed by ^3^H-TdR (**a**). Cells were lysed with RIPA lysis buffer, followed by SDS–PAGE. The specimens were then subjected to Western blotting analysis. The protein levels of Ki67, cyclin D1, p21, and PCNA were monitored by Gel DocTM XR imaging system (**b**). The corresponding quantitative analysis was also carried out by Image J software (**c**). For cell apoptosis assay, about 10 μl Annexin V-FITC and 5 μl PI was introduced. Flow cytometry was used to detect cell apoptosis (**d, e**). The caspase-3 activity was also determined using a commercial kit to assess cell apoptosis (**f**). *p < 0.05. *MSC* mesenchymal stem cell, *NC* miR-control, *PCNA* proliferating cell nuclear antigen

Uncontrolled PASMC proliferation and resistance to apoptosis contribute to vascular remodeling and are widely accepted as the major pathological features of PAH [1, 7]. To further elucidate the mechanism underlying the MSC-let-7a-mediated protective effect against PAH, we analyzed the MSC-let-7a function in PASMC proliferation. As expected, exposure to hypoxia evoked PASMC proliferation by approximately 1.89 times (Fig. 4a). However, co-culture with MSCs significantly inhibited hypoxia-induced PASMC proliferation, which was further augmented when PASMCs were co-cultured with let-7a-overexpressed MSCs. Additionally, hypoxia-triggered expression of known proliferation markers Ki67 and PCNA were also dramatically dampened after MSC-let-7a treatment (Fig. 4b and c). Simultaneously, MSC-let-7a treatment mitigated cyclin D1 levels in hypoxia-exposed PASMCs, concomitant with the increase in the cell cycle inhibitor protein p21.

### MSC-let-7a administration abrogates apoptosis resistance of PASMC

As shown in Fig. 4d, MSC treatment deteriorated PASMC cell apoptosis upon hypoxia. A higher apoptotic rate of PASMCs was validated after co-culture with let-7a-elevated MSCs and reached 31.2 ± 1.04% (Fig. 4e). The increase in caspase 3 activity further identified the enhanced impact of MSC-let-7a on PASMC apoptotic response, in contrast to the MSC-treated group (Fig. 4f). Thus, MSC-let-7a could suppress PASMC growth by dampening cell proliferation and antagonizing cell resistance to apoptosis.

### STAT3-BMPR2 signaling is involved in the growth inhibition of PASMCs triggered with MSC-let-7a

**Fig. 5:**
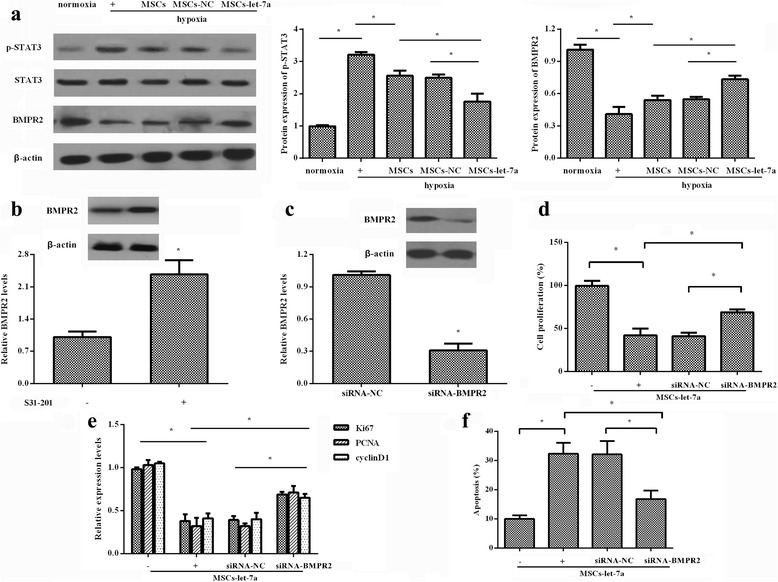
The STAT3-BMPR2 signaling was responsible for MSC-let-7a-regulated growth inhibition of PASMCs. After co-culture with MSCs modified with let-7a or NC, the expression levels of STAT3, p-STAT3, and downstream BMPR2 were determined in PASMCs upon hypoxia by Western blotting. Cells cultured under normoxia were defined as the control group (**a**). Following treatment with the STAT3 inhibitor S31-201 (15 μM), the protein levels of BMPR2 in PASMCs were analyzed (**b**). Following preconditioning with BMPR2 siRNA for 24 h, the protein level of BMPR2 was determined by Western blotting (**c**). PASMCs were treated with BMPR2 siRNA and co-cultured with or without MSC-let-7a. Cell proliferation (**d**) and proliferation-related gene expression (including Ki67, cyclin D1, and PCNA) (**e**) were then determined. (**f**) Cell apoptosis was assessed by flow cytometry. *p < 0.05. *BMPR2* bone morphogenetic protein receptor 2, *MSC* mesenchymal stem cell, *NC* miR-control, *PCNA* proliferating cell nuclear antigen, *siRNA* small interfering RNA, *STAT3* signal transducers and activators of transcription 3

The activation of the STAT3-BMPR2 pathway has been documented in PAH animal models and can regulate smooth muscle cell (SMC) growth [17]. To elucidate the underlying mechanism involved in MSC-let-7a-mediated inhibition of PASMC growth, the activation of STAT3 was determined. As shown in Fig. 5a, MSC-let-7a abrogated hypoxia-induced expression of p-STAT3 and increased downstream BMPR2 expression, suggesting that MSC-let-7a abolished the activation of the STAT3-BMPR2 pathway. When stopping STAT3 signaling with its inhibitor S31-201, the expression of BMPR2 was enhanced (Fig. 5b). Furthermore, cessation of BMPR2 expression was increased in PASMCs following BMPR2 siRNA transfection (Fig. 5c). Notably, the suppressive role of MSC-let-7a in SMC proliferation was also restored when cells were pre-administered with BMPR2 siRNA (Fig. 5d). Moreover, the corresponding downregulation in the expression of cell proliferation-related molecules, including ki67, cyclin D1, and PCNA, was also ameliorated (Fig. 5e). Furthermore, suppressing BMPR2 expression remarkably counteracted the pro-apoptotic effect of MSC-let-7a on PASMCs (Fig. 5f). Taken together, these data indicated that MSC-let-7a might regulate PASMC growth majorly by the STAT3-BMPR2 pathway.

## Discussion

Recently, let-7a has generated broad interest as a promising therapeutic agent for several diseases associated with proliferative and metabolic abnormalities, including cancer [24]. A previous study corroborated the downregulation of let-7a in lung injury [25]. Interestingly, its decrease has also been validated in PAH animal models and patients [16, 19]. In this study, depression of let-7a was observed in PAH rats. Moreover, MSC treatment enhanced let-7a expression in the lungs of PAH rats, indicating a potential role of let-7a in MSC-mediated therapy for PAH. Concomitantly, we also simulated the condition of PAH by hypoxia exposure in vitro and observed a high expression of let-7a in hypoxia-treated MSCs, suggesting that MSCs could induce the expression of let-7a in the progression of PAH. Next, we explored the effect of let-7a-modified MSCs on PAH. As expected, we successfully detected the retention of MSCs in lung tissues. Importantly, let-7a-MSC treatment obviously suppressed RVSP and RVH, which is the final pathway leading to the death of patients [1]. Furthermore, application of MSC-let-7a also attenuated the wall thickness of pulmonary arterioles and restrained the expression of ECM protein Fn and α-SMA, indicating an inhibitory effect of MSC-let-7a on vascular remodeling. Importantly, the MSCs modified with let-7a exhibited more favorable effects in ameliorating MCT-induced PAH compared to the MSC-only groups, indicating a promising potential therapeutic role of MSC-let-7a in PAH.

Excessive proliferation and apoptosis resistance of PASMCs are major pathological features of PAH and rank as key events in the pathogenesis of PAH [7]. Furthermore, PASMC proliferation and migration also facilitate the remodeling of the pulmonary artery, which is a vital element of current PAH therapies [26]. Hypoxia is widely accepted as a trigger of pulmonary vascular remodeling and the subsequent pulmonary hypertension. To elucidate the underlying mechanism involved in the MSC-let-7a-mediated protective effect against PAH, the proliferation and apoptosis of PASMCs were investigated. Here, hypoxia exposure significantly induced the proliferation of PASMCs, as supported by previous reports [27]. Nevertheless, this increase was mitigated when cells were incubated with MSC-let-7a. Moreover, MSC-let-7a treatment remarkably attenuated cell resistance to apoptosis. Therefore, these results confirmed that MSC-let-7a suppressed the growth of PASMCs by suppressing cell proliferation and cell resistance to apoptosis, which may ameliorate the progression of PAH.

Signal transducers and activators of transcription 3 (STAT3) signaling exerts important roles in various physiopathological processes, such as angiogenesis, immune response, and cellular functions [28, 29]. Recent research corroborated the abnormal activation of STAT3 signaling in PAH patients [17]. Moreover, the increase in the pro-proliferative phenotype of PASMCs has been shown to be associated with the activation of the STAT3-BMPR2 pathway [17, 30–32]. Emerging studies suggest that let-7a may restrain tumor cell proliferation by blocking the STAT3 pathway [28, 33]. Accordingly, to elucidate the mechanism involved in the inhibitory effect of MSC-let-7a on PASMC growth, STAT3 signaling was evaluated. Consistent with our previous hypothesis, MSC-let-7a significantly antagonized p-STAT3 levels, concomitant with the upregulation in its downstream BMPR2. When blocking the STAT3 signaling with S31-201, an antagonist of the STAT3 pathway, BMPR2 expression was increased. As a surface protein receptor of BMPs, BMPR2 can be expressed on vascular SMCs and identified as a well-established inhibitor of vascular SMC proliferation, but it provokes cell death [31, 34]. Interestingly, suppression of PASMC proliferation and enhancement of cell apoptosis triggered by MSC-let-7a were obviously abated when silencing BMPR2 expression. Therefore, these data suggest that STAT3-BMPR2 signaling might account for MSC-let-7a-regulated PASMC survival. However, blocking BMPR2 levels could not absolutely abolish the effect of MSC-let-7a on PASMC growth. Whether other molecules or pathways are involved in this process will be explored in our future research.

## Conclusions

This study indicated that MSC-let-7a could ameliorate the development of MCT-induced PAH by suppressing the growth of PASMCs. Furthermore, the STAT3-BMPR2 pathway was involved in MSC-let-7a-mediated PASMC proliferation. As a whole, our research may illustrate the protective role of MSC-let-7a in the progression of PAH. Therefore, this study provides the groundwork for the further study of MSC-let-7a as a therapeutic strategy against PAH. Thus, it may provide a promising therapeutic approach for PAH. However, further studies will explore the above mechanism in vivo. This study only clarifies the effect of MSC-let-7a on PAH in an animal model. There is still a long way to go to investigate the application of MSC-let-7a in human therapy, which will be the focus of our future research. Futhermore, recent research has also corroborated the critical implication of lipids, especially the oxidized lipids, in the pathophysiological hallmarks of PAH, such as SMC proliferation [35]. Interestingly, a previous study also corroborated the protective effect of let-7a on oxidized low-density lipoprotein-induced endothelial cell injury [36]. Whether MSC-let-7a can attenuate the development of PAH by regulating lipid metabolism will provide a new direction for further research and will be explored in our future research.

## Abbreviations

BMPR2: Bone morphogenetic protein receptor 2
BW: Body weight
ECM: Extracellular matrix
FBS: Fetal bovine serum
FITC: Fluorescein isothiocyanate
Fn: Fibronectin
GFP: Green fluorescent protein
LV: Left ventricular
MCT: Monocrotaline
miRNA: MicroRNA
MSC: Mesenchymal stem cell
NC: miR-control
P: Passage
PAH: Pulmonary arterial hypertension
PASMC: Pulmonary artery smooth muscle cell
PBS: Phosphate-buffered saline
PCNA: Proliferating cell nuclear antigen
rMSC: Rat mesenchymal stem cell
rPASMC: Rat pulmonary artery smooth muscle cell
RV: Right ventricular
RVH: Right ventricular hypertrophy
RVSP: Right ventricular systolic pressure
RVW: Right ventricle weight
Sep: Septum
siRNA: Small interfering RNA
SMA: Smooth muscle actin
SMC: Smooth muscle cell
STAT3: Signal transducers and activators of transcription 3
